# Resveratrol: A Focus on Several Neurodegenerative Diseases

**DOI:** 10.1155/2015/392169

**Published:** 2015-06-09

**Authors:** Ester Tellone, Antonio Galtieri, Annamaria Russo, Bruno Giardina, Silvana Ficarra

**Affiliations:** ^1^Department of Chemical Sciences, University of Messina, V. le Ferdinando Stagno d'Alcontres 31, 98166 Messina, Italy; ^2^Biochemistry and Clinical Biochemistry Institute, School of Medicine, Catholic University, L. go F. Vito n.1, 00168 Rome, Italy; ^3^C.N.R. Institute of Chemistry of Molecular Recognition, L. go F. Vito n.1, 00168 Rome, Italy

## Abstract

Molecules of the plant world are proving their effectiveness in countering, slowing down, and regressing many diseases. The resveratrol for its intrinsic properties related to its stilbene structure has been proven to be a universal panacea, especially for a wide range of neurodegenerative diseases. This paper evaluates (in vivo and in vitro) the various molecular targets of this peculiar polyphenol and its ability to effectively counter several neurodegenerative disorders such as Parkinson's, Alzheimer's, and Huntington's diseases and amyotrophic lateral sclerosis. What emerges is that, in the deep heterogeneity of the pathologies evaluated, resveratrol through a convergence on the protein targets is able to give therapeutic responses in neuronal cells deeply diversified not only in morphological structure but especially in their function performed in the anatomical district to which they belong.

## 1. Introduction

**Figure 1:**
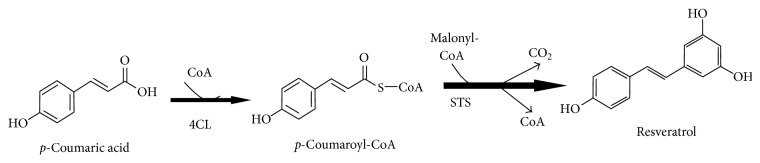
Biosynthetic pathway of RV.

Resveratrol (RV), or 3,5,4′-trihydroxy-trans-stilbene, is an antifungal molecule of the stilbene family produced in a variety of plant species in response to pathogen attack or under stress conditions such as UV radiation and exposure to heavy metal ions [1]. It is a natural phenol found in red grapes, mulberries, peanuts, wines, and tea and it can be extracted from red wine during fermentation of grape skin. RV exists in two isoforms: trans-RV, the more stable form, and cis-isomer, both produced as a branch from the phenylpropanoid pathway [2]. In plants, RV biosynthesis starts by the coupling of* p-*coumaric acid, an intermediate in lignin production, to coenzyme A (CoA) by the action of 4-coumarate CoA ligase (4CL); see Figure 1. Subsequently, coumaroyl-CoA is converted into RV with release of carbon dioxide, by sequential addition of three units of malonyl-CoA by the action of stilbene synthase (STS) [3].

In in vitro and in vivo experiments, RV displays a wide range of beneficial effects on human diseases but the mechanisms by which RV exerts its action have not yet been clarified. After oral administration, RV is transported to the circulatory system and is distributed to all organs where it remains detectable for some hours after administration; it can also rapidly cross the blood-brain enriching the brain tissue [4]. However, one of the main limitations of this drug is its low oral bioavailability, due to rapid excretion and extensive metabolism into variants of glucuronide and sulfonated conjugates of unknown potential biological activities [5].

RV shows several mechanisms of action and interacts with a significant number of molecular targets, but its positive effect on the human health seems to be related mainly to its antioxidant activity. Since oxidative stress appears to be closely related to major neuronal pathologies, RV treatment has been tested with positive results in neurodegenerative disorders such as Alzheimer's disease (AD), Huntington's disease (HD), prion, cerebral ischemia, Parkinson's disease (PD), epilepsy, and amyotrophic lateral sclerosis (ALS) [6], but there are quite a few studies to describe the dose dependency of the drug towards these health benefits.

This review aims to give an overview of the beneficial effects of RV on several human neurodegenerations as AD, PD, HD and ALS trying to highlight the mechanisms by which the polyphenol exerts its specific activity.

### 1.1. Oxidative Stress and Neurodegenerative Diseases

**Figure 2:**
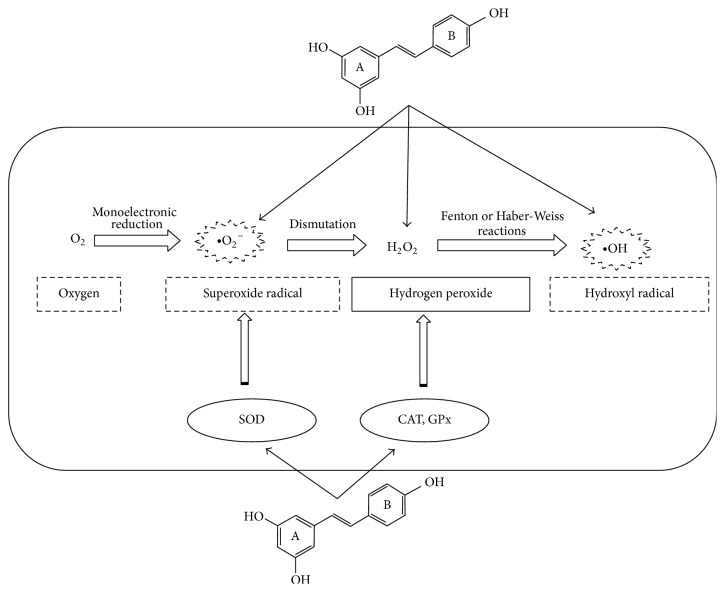
Antioxidant activity of RV against ROS.

A suitable amount of free radicals is essential for life because they are involved in cell signaling and are used by phagocytes for their bactericidal action [7]. However, nonessential production of reactive oxygen species (ROS) is suggested to be strongly associated with the aging process and certain degenerative disorders. So to human health, the balance is very important between free radicals produced by metabolism or derived from environmental sources and the antioxidant defense systems such as superoxide dismutase (SOD), catalase (CAT), and glutathione peroxidase (GPx) able to promptly scavenge and neutralize free radicals (Figure 2).

Among the pathologies linked to oxidative stress, the neurodegenerative disorders occupy a relevant side because neurons are particularly vulnerable to attack by free radical attacks and oxidative stress is one of the major pathogenic mechanisms in the etiology of a variety of late onset diseases. The high vulnerability of the nervous system, including the brain, spinal cord, and peripheral nerves, to oxidative stress is due to its elevated bioenergetics and oxygen requirements. In fact neurons with axons and multiple synapses have high ATP demand and they are largely responsible for the brain's massive consumption of oxygen in the respiratory chain; this coupled with the high content of lipid and easily mobilizable iron from several areas of the brain can stimulate the generation of ROS (Fenton and/or Haber-Weiss reactions) [8]. Since age is related to the reduced capacity to counteract oxidative stress, also this can lead to irreversible damages that can contribute to the pathogenesis of neurodegenerative disorders. At the pathological level, almost all neurodegenerative diseases share common features such as the generation of misfolded protein deposits, metal ion deregulation, and exposure to oxidative stress [9–13]. Generally, the protein aggregates are primarily composed of typical proteins in different diseases. For example, in AD the extracellular senile plaques are predominantly consisted of amyloid-*β* (A*β*) peptides derived from the mutations in genes encoding the amyloid precursor protein (APP), while the intracellular tangles are from hyperphosphorylated Tau protein; HD is caused by the gene mutation that affects the conformation and aggregation propensity of the huntingtin protein (htt) [14]; in PD the accumulation of intracytoplasmic Lewy bodies is mainly composed of *α*-synuclein and ubiquitin [15]; similarly the protein products of the associated diverse set of genes including SOD1, TDP43, FUS, UBQLN2, and C9OKF72 have also been found in neuronal aggregates from ALS patients [16–18].

Besides, iron changes have been detected in multiple sclerosis, spastic paraplegia, and ALS, reinforcing the belief that iron accumulation is a secondary alteration associated with neurodegeneration, probably due to the changes in the integrity of the blood brain caused by abnormal vascularization of tissue or by inflammatory events [19].

**Figure 3:**
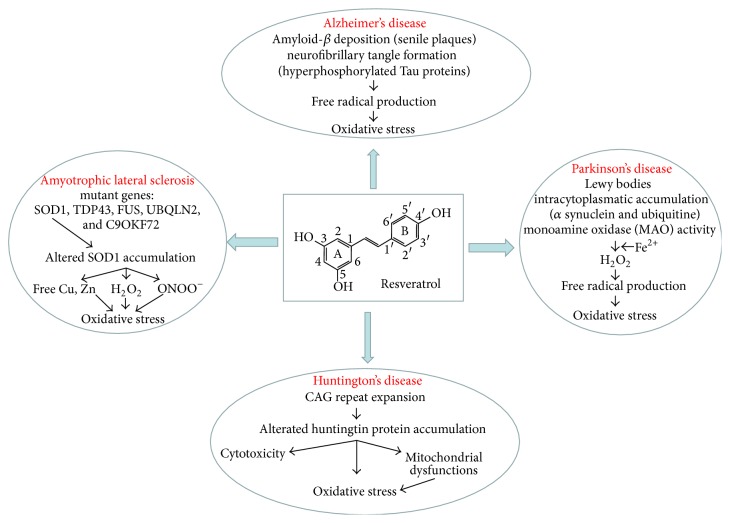
RV and neurodegenerative disease.

It is demonstrated yet that markers of oxidative stress precede pathologic lesions in AD, including senile plaques and neurofibrillary tangles [20]. Furthermore, ATP depletion or lipid and protein peroxidation induced by ROS is also implicated in PD and kills neurons by necrotic processes [21]; protein oxidative damage in the form of protein carbonyls and increased levels of 8-hydroxydeoxyguanosine are also present in PD brain and some evidences suggest a role for nitration and nitrosylation of certain proteins due to reactive nitrogen species [22, 23]. In this context, the linkage between neurodegenerative diseases and oxidative stress is largely investigated by researchers. Support for this curiosity comes from increasing attention to the efficacy of therapies with antioxidants and to the scavenger substances as protectors of nervous tissue from damage by oxidative stress. Clearly, strategies aimed at limiting free radical production, oxidative stress, and damage may slow the progression of neurodegenerative diseases. Actually there is a great interest to study the neuroprotective effects of natural products obtained from plants. There are several natural compounds with antioxidant properties which may contribute to counteracting oxidative stress by working to neutralize the excess free radicals and stopping them from starting the chain reactions that contribute to disease. In this context, RV for its chemical properties may be a very promising lead compound to counteract neuronal pathologies (Figure 3).

### 1.2. Antioxidant and Prooxidant Effects of RV

The antioxidant and prooxidant activities of RV appear to be dose and cell type dependent. In particular, antioxidant properties of RV seem to be enhanced with increasing concentration of the drug and Cavallaro et al. [24] demonstrated that RV inhibited superoxide anion generation both in low and high concentrations.

RV's activity as antioxidant and free radical scavenger is related to its ability to transfer hydrogen atoms or electrons to the free radicals [25, 26]. In this context, the characteristic position of hydroxyl groups plays a major role, among which the 4′-hydroxyl group is the most reactive one [27, 28]. RV antioxidant properties result from its chemical structure; in fact the molecule contains two phenol groups in which the presence of conjugated double bond makes the electrons more delocalized. Support of this hypothetic mechanism of action comes from studies on oxyresveratrol (oxy-RV), demonstrating in the modified drug a more effective antioxidant activity than RV probably due to the extra hydroxyl group on oxy-RV which makes oxy-RV a better hydrogen donor enhancing its antioxidant activity [29]. So RV may donate hydrogen to free radicals inhibiting the peroxidation and protecting cellular DNA, lipids, and proteins from oxidative damage.

Recently, using planar lipid bilayer and liposome models, it has been shown that RV at low doses interacts with the surface polar groups and at higher doses localizes in the outer leaflet of the lipid bilayer. Interestingly, RV localization is strictly related to the antioxidant properties of the drug, because the polyphenol localization in the membrane bilayer prevents lipid peroxidation [30] and intraerythrocyte RV, by interacting with hemoglobin, may protect the protein against oxidative damage [31]. The drug breaks the chain-reaction process of lipid peroxidation by scavenging free radicals and forming phenoxy radicals that are stabilized by resonance. For the global reactivity of RV toward ^∙^OH radical, the most electrophilic radical is the sequential electron proton transfer (SEPT): RV + ^∙^OH → RV^+∙^ + OH^−^↔RV(−H)^∙^ + H_2_O [26].

**Figure 4:**
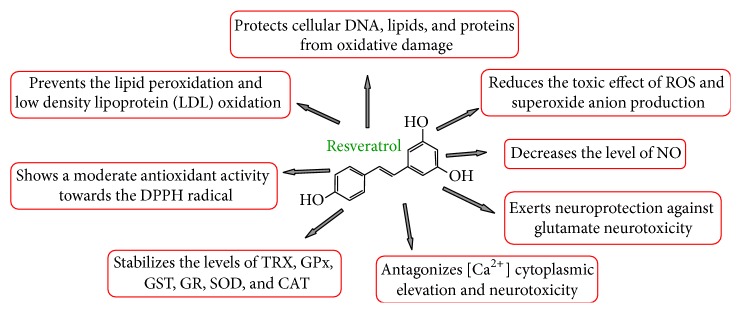
Main effects of RV.

RV shows a moderate antioxidant activity towards the 1,1-diphenyl-2-picrylhydrazyl (DPPH) radical, induces a significant reduction of superoxide anion, and decreases oxidation of hemoglobin, contributing to decreasing the superoxide concentration [31, 32]. In particular, RV action against oxidation of hemoglobin may be due to the action of its phenol groups which are able to reduce Fe^3+^ to Fe^2+^. It is also known that RV prevents low density lipoprotein (LDL) oxidation, responsible of atheromatous plaques in atherosclerosis disease [33]; in fact RV was shown to be more potent than flavonoids in preventing copper-catalyzed oxidation [34] and contributed to maintaining the levels of antioxidant enzymes like GPx, glutathione-S-transferase (GST) and reductase (GR), SOD, and CAT [35, 36]. Most likely part of beneficial properties of RV is probably related to concomitant downregulation of the expression of inducible NO synthase (iNOS) and upregulation of vasorelaxant endothelial NO synthase (eNOS) as observed by several studies [37–39]. Interestingly, RV acting as an antioxidant prevents the formation of toxic A*β* oligomers and protofibrillar intermediates, delaying the induced A*β* toxicity in different neuronal culture models [40]. These studies contributed to shedding light on the molecular mechanisms potentially involved in the beneficial effect of RV intake against AD (Figure 4) [41].

On the other hand, Ahmad results suggest prooxidant properties of RV at low concentration due to an increase in intracellular superoxide production and in the presence of copper ions [42, 43]. In detail, RV promotes the reduction of copper (II) to copper (I) [36] but its binding with copper promotes prooxidant activity of the drug [44].

### 1.3. RV Molecular Targets

Although the interest on RV was initially focused on its antioxidant properties, it has been reported that the drug affects a wide range of signaling transduction pathways.

Several studies using both in vitro and in vivo model systems have illustrated RV capacity to modulate a multitude of biological activity associated with cellular growth and differentiation, apoptosis, angiogenesis, and metastasis [45–47]. Thus, RV modulates multiple signaling pathways that interrupt the carcinogenic process and is also able to extend one or more stages of this process. Also, RV has been shown to inhibit a plethora of enzymes belonging to different classes, including (but not limited to) kinases, lipo- and cyclooxygenases, sirtuins, and other proteins. Furthermore, RV is reported to induce cell cycle arrest in many cancer cell lines, probably through the modulation of cyclin dependent kinase (CDK) associated proteins and through the activity of the tumor suppressor protein p53 dependent and independent pathways [48–50]. p53 is a key mediator in the prevention of carcinogenesis because it is involved in the regulation of cell proliferation and apoptosis [51].

In addition, RV has been shown to mediate the activation of sirtuin-1 (SIRT1). Sirtuin enzymes are a family of highly conserved deacetylase proteins with potential therapeutic targets in a variety of human diseases including diabetes, inflammatory disorders, and neurodegenerative diseases [18].

RV antagonizes calcium cytoplasmic elevation and neurotoxicity generated by ASL [52, 53] and shows many antioxidant properties. RV has been proven to exert neuroprotection against glutamate toxicity in neuronal cultures [54] and through P13K/Akt pathway by downregulating the expression of glycogen synthase kinase 3 (GSK-3*β*) [55]. GSK-3*β* is involved in multiple signaling pathways and has several phosphorylation targets; it is mainly localized in the cytosol, but lower amounts are expressed in the nucleus and mitochondria, where it has a regulatory role in the cell death pathway elicited by stress conditions [56, 57]. A number of studies on cerebral blood flow (CBF) and cognitive performance in humans provide evidences that RV administration can modulate brain functions improving glucose metabolism [58] and vasorelaxation by promoting eNOS and/or NO synthesis [59, 60].

RV positively influences telomeres length promoting the expression of Werner syndrome ATP-dependent-helicase, a telomere maintenance factor [61, 62]; this protection is important for mitochondrial efficiency and oxidative stress defenses because telomere shortening, activating p53 represses the transcription of the peroxisome proliferator-activated receptor gamma coactivator-1 alpha (PGC-1*α*) and impairs mitochondrial function [63]. But RV stimulates PGC-1*α* also through its interaction with SIRT1 where deacetylating activates PGC-1*α*. PGC-1*α* is a potent stimulator of mitochondrial biogenesis and respiration because it induces the transcription of nuclear respiratory factor (NRF)1 and NRF2, leading to the increased expression of mitochondrial transcription factor A (mtTFA) [64] as well as other nuclear-encoded mitochondria subunits of the electron transport chain complex [65].

RV increased cAMP and modulated Akt pathway in cell model studies [66]; besides, RV activates AMP protein kinase-SIRT1 autophagy pathway in PD cell model studies [67], upregulates antiapoptotic Bcl-2 protein, and downregulates Bax protein expression [68] and also acts as mitochondrial antioxidant by elevating the levels of antioxidants thioredoxin-2 (TRX2) and X-chromosome-linked inhibitor of apoptosis protein [69]. Another study has shown that RV increased expression of Bcl-2, thus preventing neuronal apoptosis [70]. RV appears to be effective in reducing the inflammatory status; the drug attenuates the activation of immune cells and subsequent synthesis and release of inflammatory mediators through the inhibition of transcription factors such as nuclear factor-kappaB (NF-*κ*B) [71].

## 2. RV and Alzheimer's Disease

AD is a progressive, age dependent neurodegenerative disorder leading to the most common form of dementia in elderly people. Histopathological studies of the AD brain revealed in the cortex and hippocampus the presence of ultrastructural changes triggered by two classical lesions, the extracellular senile plaques mainly composed of A*β* peptides and intracellular neurofibrillary tangles composed of hyperphosphorylated Tau proteins [72, 73]. Tau is a multifunctional microtubule-associated protein that plays major role in assembly of microtubules and in bridging these polymers with other cytoskeletal filaments [74]. The earliest modification found in AD brains consists of hyperphosphorylation on Tau by the action of different protein kinase and phosphatase systems that lead to structural and conformational changes in this protein, affecting its binding with tubulin and the capacity to promote microtubule assembly [75]. The most relevant protein kinases involved in Tau modification in neurofibrillary degeneration are GSK3*β* [76]. GSK3*β* would increase Tau hyperphosphorylation at sites that transform Tau into a protein lacking the ability to associate with cytoskeleton.

Although most of the AD cases are sporadic with an obscure etiology, some forms are inherited and several genes encoding APP, presenilin 1 (PS1), and presenilin 2 (PS2) were found to be implicated in familial forms of the disease. In both cases (familial and sporadic) A*β* peptides were regarded as a causative event in the pathogenesis of the APP by *β* and *γ* secretases. The formation of diffusible A*β* oligomers that can aggregate and form fibril and amyloid deposition plaques is a process that initiates the synaptic malfunction and the AD toxic effects [77]. Neuropathologic studies show an increased rate of apoptotic neurons in postmortem sample from AD patients [78]. Apoptosis, is due to a number of cascades of cellular events involving caspase activation that actively kills the cell. In the nervous system, apoptosis appears to be triggered by trophic factor deprivation. The lack of activation of intracellular pathway transducing trophic factor leads to caspase activation. Trophic factor deprivation in neurons may result in dephosphorylation of BAD that interacts with Bcl-2 facilitating the release from mitochondria of cytochrome C and apoptosis-activating factor (apaf) which finally leads to caspase 3 activation [79]. These findings in motor neurons induce oxidative stress involving the production of nitric oxide, superoxide, and peroxynitrite which also activate caspase 3, suggesting a more general role of the oxidants as mediator of apoptosis. At this regard, we should not forget that also presenilins seem to play a role as modulators of neuronal apoptosis too. RV has been shown to inhibit A*β* fibrils formation [80, 81] by degreasing A*β* production through sirtuin dependent activation; the drug potentiates SIRT1 activity via an allosteric mechanism [82, 83]. In detail, neuronal SIRT1 expression decreased levels of ROCK1, a serine/threonine Rho-kinase previously shown to regulate A*β* metabolism and this effect enhanced *α*-secretase activity, an enzyme which process APP along a nonamyloidogenic pathway [84, 85]. Additionally in vitro observations indicated that SIRT1 can directly deacetylate Tau protein at multiple residues. The removal of these acetyl groups may expose Lys residues to ubiquitin ligases so that Tau protein could be marked for proteasomal degradation [86, 87].

**Figure 5:**
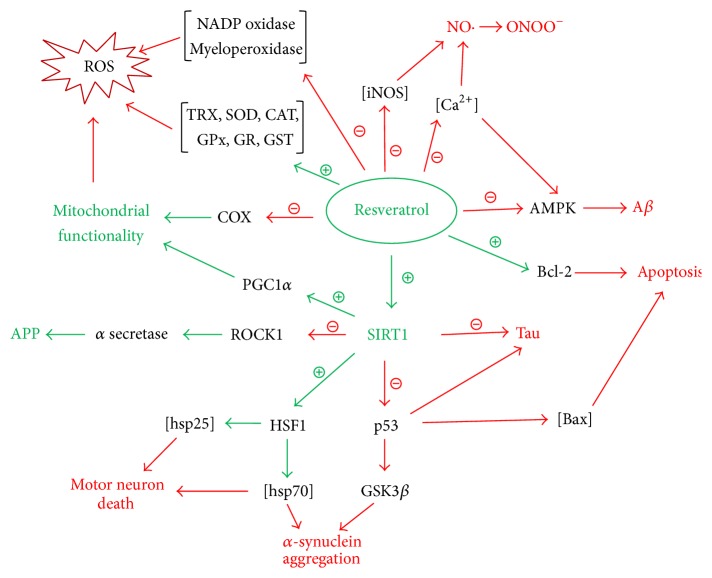
Global representation of RV targets in neurodegenerative protection.

Hooper et al. [88] reported that p53 is upregulated approximately 2-fold in the superior temporal gyrus of AD and that p53 induces Tau indirect phosphorylation. Thus, p53 seems to play a pivotal role in AD implying that modulation of cell death pathways might be of therapeutic benefit (and indeed in other age related neurological disorders). The identification of p53 as a SIRT1 substrate highlights a further protective role of RV in AD-related cognitive decline. In fact, allosteric modulation of RV on SIRT1 activating deacetylation of p53 attenuates its activity [89]. Additionally, inhibition of p53 by RV might alter and in some way partially inhibit the GSK3*β* and p53 interaction. Since p53 and GSK3*β* are both involved in the apoptotic pathway (GSK3*β* overactivity leads to increased levels of plaques and tangles and p53 activity induces Tau phosphorylation), a strong RV effect may be speculated on AD against several molecular targets. Besides, Vingtdeux et al. [90] demonstrated the antiamyloidogenic effect of RV through activation of AMP-protein kinase (AMPK). AMPK is a heterotrimeric Ser/Thr protein kinase activated by different upstream kinases among which calcium/calmodulin-dependent protein kinase kinase-*β* (CamKK*β*) is predominantly expressed in neuronal tissue [91]. AMPK signaling controls A*β* metabolism and RV increasing intracellular calcium levels promote AMPK activation by the CamKK*β* pathway [90, 92–94]. Alterations of mitochondrial functioning followed by ROS generation are two alarming conditions known in aging and early stages of AD [95]. RV efficiently counteracts both pathological conditions, on the one hand through activation of SIRT1 and the PGC1*α* pathway that lead to improved mitochondrial function and efficiency and on the other hand through its antioxidant activity reducing ROS generation [31, 32, 96–98] (Figure 5).

## 3. RV and Huntington's Disease

HD is an autosomal-dominant neurological disorder; the most striking pathological manifestation of the disease is a gradual loss of neurons predominantly in the striatum causing motor abnormalities and cognitive decline [99]. HD genesis is caused by an unstable trinucleotide CAG repeat expansion at the N-terminus of the gene encoding htt [100]. The mutation leads to the production of the htt with an abnormal protein-protein interaction named mutant polyglutamine htt (m-htt) which forms cytotoxic aggregates in neurons [101, 102]. Overexpression of htt fragment in neurons results in a gain of function mechanosensory defect that is the cause of the HD pathology.

RV beneficial effects against 3-nitropropionic acid suggest a role of the drug in protecting by neurotoxins in HD because 3-nitropropionic acid is an inhibitor of complex II of the electron transport chain, which causes HD's like symptoms. RV inhibits cyclooxygenase I (COX) activity significantly improving motor and cognitive impairments in the 3-nitropropionic acid-induced model of HD [103]. In addition, RV protects neurons against cytotoxicity of the mutant polyglutamine htt acting through SIRT1 activation [104]. Several mechanisms have been proposed by which m-htt may trigger striatal neurodegeneration, including mitochondrial dysfunction, oxidative stress, and apoptosis. In this context, p53 activation plays a crucial role in mediating m-htt toxic effects in human neuronal cells. The tumor suppressor p53 mediates dysfunctions and cytotoxicity in HD cells and in transgenic mouse whereas its inhibition prevents these phenotypes [105]. RV protects cells by toxic effects of m-htt potentiating SIRT1 activity and inducing an indirect inhibition of p53 because SIRT1 interacts with and deacetylates p53 [106, 107]. The deacetylation of p53 attenuates its activity and inhibits p53 dependent apoptosis. In general, p53 activation which happens in HD has been linked to enhanced mitochondrial oxidation [108, 109], while activation of SIRT1 as happens in presence of RV allows the cell to adapt to situations of energy stress [89].

RV can effectively interject in the mitochondrial oxidation through its antioxidant properties and counteract impaired mitochondrial function through the activation of the SIRT1-PGC1*α* pathway [110–112]. In fact, PGC1*α* regulates the expression and activities of ROS scavenging antioxidant enzymes and therefore counteracts oxidative stress [113].

## 4. RV and Parkinson's Disease

PD is the second most common neurodegenerative disorder after AD, affecting nearly 2% of individuals over the age of 65 in industrialized countries [114]. Although the etiology of sporadic PD is poorly understood, there is evidence that both environmental factors and genetic predisposition contribute to its development. Rare missense mutations and more frequent multiplications of a large genomic region including the *α*-synuclein gene cause autosomal dominant Parkinsonian syndromes [115]. Clinically, PD is characterized by a progressive neurodegenerative disorders showing invalidating neurological symptoms: increasing muscle rigidity, tremor, bradykinesia, and in extreme cases a nearly complete loss of movements. Motor symptoms originate from the degeneration of dopaminergic neurons of the substantia nigra with a consequent loss of dopamine and accumulation of intracytoplasmic Lewy bodies, inclusions that contain *α*-synuclein and ubiquitin [15]. Dopamine is inactivated by the monoamine oxidase enzyme (MAO), a reaction that yields significant amounts of hydrogen peroxide that must be continuously detoxified by intracellular antioxidants.

Dopaminergic cells are believed to die by apoptosis rather than necrosis, but even this basic concept is disputed [116]; there is no doubt that oxidative and nitrative stress occurring in substantia nigra is prominent features of this disease [117].

The source of nitrogen species (nitric oxide and peroxynitrite) is clearly related to alterations in iNOS activity. The origin of oxygen radicals is much less clear and is based mainly on indirect biochemical changes, such as increased iron levels, alterations in antioxidant mechanisms, and mitochondrial dysfunction.

The involvement of mitochondrial impairment in PD pathogenesis has been established for over two decades. Complex I inhibition is known to be the major source of free radicals, and it is thought that the alteration in its functionally could, above and beyond the declining production of ATP, give rise to increased oxidative stress, thus explaining the emergence of the disease [118].

RV treatment ameliorates the mitochondrial respiratory capacities via a pathway in which SIRT1-AMPK and PGC-1*α* play a pivotal role. In detail, the activation of AMPK-SIRT1 signaling by RV results in the induction of the PGC-1*α* activity [119]. The impact of PGC-1*α* activation on mitochondrial respiratory capacities leads to an increase of mitochondrial biogenesis and improves mitochondrial function.

An interesting hypothesis for the vulnerability of certain neuronal groups in PD is the relation between the decline in ATP and the calcium intracellular oscillations. MAO induced metabolism of dopamine produces calcium signaling in astrocytes through ROS (hydrogen peroxide, principally) [120]. This creates a metabolic stress because the repeated and persistent entry of calcium into cells needs to be counterbalanced by ATP demanding pumps to restore the calcium homeostasis [23]. It has been demonstrated that the opening of L-type calcium channels in the mitochondria of such neurons makes them highly vulnerable to disease process [121]. RV can effectively interject the progression of PD preventing calcium elevation [52, 122, 123].

In experimental models of PD, treatment with RV exerts neuroprotective effects on dopaminergic neurons probably related to antioxidant properties of the drug [118, 124]. In this context, the RV scavenger activity against hydrogen peroxide (H_2_O_2_) may be particularly efficient; the drug at 100 *μ*g/mL exhibited 60% of its effect [25]. Besides, RV inducing activation and expression of SIRT1 protects against pathological *α*-synuclein aggregation [125]; in detail, SIRT1 can deacetylate and activate heat shock factor 1 (HSF1), which affects transcription of molecular chaperons including heat shock proteins 70 (hsp70). Hsp70 regulate homeostasis of cellular proteins decreasing the formation of abnormal protein aggregates [18, 126, 127].

Studies have shown that GSK-3*β* inhibition protects the dopaminergic neurons from various stress-induced injuries, indicating the involvement of GSK-3*β* in PD pathogenesis because *α*-synuclein is a substrate for GSK-3*β* phosphorylation [128]. RV may decrease *α*-synuclein protein expression in cellular model of PD through its downregulation and partially inhibition of GSK-3*β* [129].

## 5. RV and Amyotrophic Lateral Sclerosis

ALS is a progressive and fatal neurodegenerative disease, characterized by the selective loss of motor neurons in brain, brainstem, and spinal cord [130]. In human patients ALS symptoms onset is varied but usually begins with muscle weakness, muscle atrophy, and spasticity leading to paralysis, respiratory insufficiency, and death with a median survival time of less than 5 years. Although the selective mechanism of motor neuron death is still unknown, two ALS forms have been identified: sporadic (SALS) with no known genetic component and familial (FALS) with a positive familial history and a genetic component [131]. Currently, several genes have been identified as possible causes of onset for FALS but curiously, although these genes control different cellular mechanisms, the progression of the disease leads inexorably to motor neuron degeneration. About 20–40% of FALS forms have one of over 150 mutations in the gene for Cu, Zn superoxide dismutase 1 (SOD1) [17], while unexpectedly mutations in the TDP-43 gene, which codes for RNA binding protein, are responsible for about 5% of both FALS and SALS [132]. Really, SALS and FALS are clinically indistinguishable suggesting a common pathogenesis of the disease; in fact, the protein products of genes associated with ALS as mutant SOD1, TDP43, or FUS were found in neuronal aggregates from ALS patients and observed to coincide with the manifestation of disease symptoms in all mouse models [16, 133] suggesting that, in addition to playing a role in FALS, these proteins may be altered also in SALS forms of the disease [134, 135]. The current consensus is that most causes may converge to the motor neuron damage typified by ALS [136], from which the most studied are the following.

*SOD1 Mutations.* SOD1 is a gene that codes for SOD, an enzyme which helps to convert superoxide radicals into less harmful molecules. If SOD is damaged, free radicals accumulation could contribute to ALS. In addition, accumulation of abnormal SOD molecules may be (the seed for large) the trigger for misfolded protein that are toxic to neurons [137].

*Glutamate Toxicity.* Under normal conditions, glutamate is an important neurotransmitter but in patients with ALS glutamate is accumulated in the synapse. These elevated levels of glutamate-mediated excitation can kill motor neurons [138].

*Oxidative Stress.* Studies have found elevated levels of oxidative stress within the central neurons system in ALS [139]. This condition causing injury of adjacent neurons promotes the propagating of the disease and may be linked to the inability of mutant SOD1 to complex Cu and Zn [10]. Diminished metal binding (by SOD1) could also enhance the release of copper and zinc and trigger metal-mediated neurotoxicity.

*Mitochondrial Dysfunction.* Studies of both human and animal neurons have found extensive mitochondrial dysfunction associated with ALS [140–142]. In such cases biochemical analyses have delineated defects in the respiratory chain complexes I and IV in muscle [143], but the main morphological damage is the presence of vacuolated mitochondria derived from a detachment between the inner and the outer membrane [144]. Several observations linked mutant SOD1 with mitochondrial damage because SOD1 has been found in the mitochondria intermembrane space, in the matrix, and in the cytosolic face of the outer membrane [145]. The presence of SOD1 protects mitochondrial functionality defending proteins from oxidation but abnormal protein aggregation of mutant SOD1 could directly damage mitochondria triggering cell death [146, 147].

*Calcium Dysregulation.* Ca^2+^ dyshomeostasis has been implicated in the pathogenesis of motoneurone death in ALS [148, 149]. Calcium accumulation in intracellular compartments can lead to an increase in the production of nitric oxide and peroxynitrite both of which could be lethal to the cell [150, 151]. Yet there is considerable evidence that calcium overload and mitochondrial abnormalities are early events in toxicity at least some SOD1 mutants [152, 153].

In this context, RV has shown several benefic effects which virtually can efficiently counteract ALS molecular targets. Mitochondrial impairment and ROS generation may be reduced by antioxidant properties of RV; in this regard, Song et al. [154] demonstrated the repression of ROS level after RV treatment in the ALS mice. Besides, as mentioned before, RV protects against mitochondrial fragmentation by the activation of PGC1*α* mediated by RV-SIRT1 interaction [18, 96, 155]. Zhao et al. reported that RV through the overexpression of PGC1*α* improved motor performance and survival in a mouse model of ALS [156].

Activation of SIRT1 by RV treatment has been shown also to decrease proteotoxic stress derived from misfolded SOD1 aggregates. The proposed mechanism is that SIRT1 activated by RV can deacetylate HSF1, inducing the transcription of molecular chaperones such as hsp70 and hsp25 and decreasing motor neuron death [18, 157, 158]. Song et al. correlated the strong inhibition effects of RV on apoptosis with the potential effects of the drug to prevent the motor neurons from degeneration in ALS [154]. In detail, RV-SIRT1 interaction mediated deacetylation and inhibition of p53 ability to induce the expression of the proapoptotic factor Bax [159].

RV has already been proven to exert neuroprotection against glutamate neurotoxicity in neuronal cultures [54] and to prevent the [Ca^2+^] elevation [52].

## 6. Conclusions

In the past few years, it has become clear that the dysfunction of mitochondrial metabolism and ROS dyshomeostasis are the main contributing factors in the progression of many neurodegenerative diseases. However, whether such events are a primary cause or consequences of the disease progression is still an unanswered question. Evaluating the safety and efficacy of small molecules which exhibit remarkable multipotent ability to control and modulate ROS, metal toxicity, and abnormal protein aggregations may be important elements in the development of new therapeutic strategies to treat neurodegenerative diseases.

RV as a multitarget compound with several neuroprotective roles represents an intriguing candidate for potential application in the treatment of neurological impairments. Particularly attractive are recent studies showing the role of RV in improved mitochondrial functions and biogenesis through SIRT1/AMPK/PGC1*α* pathway which highlight RV benefits not only limited to the antioxidant and anti-inflammatory properties.

It is right to remember the potential problems related to a possible therapeutic use of the RV, because it is not much soluble in water [160].

Despite this, RV may be considered as a very promising “model compound” [161] starting from which useful and more effective derivatives could be obtained by appropriate chemical modifications and decorations of the stilbene scaffold. Recently, it is also worth noting that piceid, a precursor of RV, exhibited higher scavenging activity against hydroxyl radicals than RV in vitro [162]. Consequently, the synthesis of analogues of the RV with improved bioavailability and solubility could help raise the number of targets affected by biological molecule and better delineate the pathways of action, opening new perspectives in the search and synthesis of novel agents to treat neurodegenerative diseases.
